# On a heuristic point of view concerning the citation distribution: introducing the Wakeby distribution

**DOI:** 10.1186/s40064-015-0821-1

**Published:** 2015-02-26

**Authors:** Yurij L Katchanov, Yulia V Markova

**Affiliations:** National Research University Higher School of Economics, 20 Myasnitskaya Ulitsa, Moscow, 101000 Russian Federation; Institute of Sociology, Russian Academy of Sciences, 24/35 b.5 Krzhizhanovskogo Ulitsa, Moscow, 117218 Russian Federation

**Keywords:** Bibliometrics, Choquet – Deny convolution equation, Citation distributions, Wakeby distribution

## Abstract

The paper proposes a heuristic approach to modeling the cumulative distribution of citations of papers in scientific journals by means of the Wakeby distribution. The Markov process of citation leading to the Wakeby distribution is analyzed using the terminal time formalism. The Wakeby distribution is derived in the paper from the simple and general inhomogeneous Choquet–Deny convolution equation for a non-probability measure. We give statistical evidence that the Wakeby distribution is a reasonable approximation of the empirical citation distributions.

**AMS Subject Classification:** 91D30; 91D99

## Introduction

The number *N*(*z*) of scientific papers that has been cited a total of *z* times is one of the most widely used and strong scientometric indicators. Alternatively, one may consider more sophisticated indicators (see, e.g., (Glänzel and Moed 2013; Leydesdorff et al. 2011, 2013; Radicchi and Castellano 2011; Waltman and van Eck 2013)), but we limit ourselves here to the case in which the underlying variables are defined as the non-negative real numbers *z* and *N*(*z*). This approach has a formal defect that can be easily recognized. As a matter of fact, *z* and *N*(*z*) assume only non-negative integer values. Yet a substantial amount of previous works on the statistical distribution of citations of scientific papers treated *z* and *N*(*z*) as continuous variables in the long-time limit of the observation period, and we pursue the same approach with this paper.

Many problems of Science are describable in terms of a probability distribution. The distribution of citations over papers is important in that it connects more theoretically grounded studies with more practical problems of scientometrics (DeBellis 2009; Moed 2005). Hence, there is a great deal of literature on the distribution of citations to papers in scientific journals. The programmatic article by Lotka 1926 in 1926 was the pioneer paper in scientometric research and continues to be much in demand (see, e.g., (Egghe and Rousseau 2012)). In 1957, Shockley achieved encouraging results (Shockley 1957). Later, in 1965, de Solla Price demonstrated that the citation distribution of scientific papers has strong skewness and heavy tail 1965, and since that time, significant effort has been invested in the study of the citation distribution. De Solla Price explained this “skew distribution” in terms of the cumulative advantage principle (de Solla Price 1976): the probability that a paper will be cited grows with the number of citations it has already received. More precisely, in terms of the probability density function *f*(·), the cumulative advantage model predicts the following distribution of citations of scientific papers

(1) $$ f(z) = \frac{B(z + m, l)}{B(m, l - 1)}.  $$

Here *z* indicates the number of citations, *B*(·,·) is the beta function, and *m*, *l* are parameters. It is important to note that the formula (1) is only valid for sufficiently long times. The continuous approximation of (1) can be analytically estimated as a power-law function for some positive number *l*

(2) $$  (z \gg z_{\min})\colon f(z) \propto Cz^{- l},  $$

where *z*_min_ means a threshold value.

One of the classic results of scientometrics is the derivation of a model in which the probability distribution (PD for short) of *z*, in its asymptotic tail, is equivalent to a power-law PD (2) (Haitun 1982; Yablonsky 1985). A possible mechanism to explain the power-law distribution is a stochastic growth process in which the citation rate of a paper is defined by the total number of received citations and the time after publication (Albert and Barabási 2002; de Solla Price 1976; Dorogovtsev et al. 2000; Golosovsky and Solomon 2013; Krapivsky et al. 2000).

The other main result is the justification of the power-law approximation of the statistical distribution of citations of scientific papers (Albarrán and Ruiz-Castillo 2011; Albarrán et al. 2011; Ausloos 2014; Brzezinski 2014; Egghe 2007, Eom and Fortunato 2011; Peterson et al. 2010; Radicchi and Castellano 2012; Redner 1998; Stringer et al 2010*; Waltman et al.*^2012^*; Zhao and Ye*^2013^*). However, the power-law distribution is possessed of a number of characteristics limiting its application (Clauset et al.*^2009^*; Golosovsky and Solomon*^2012^*; Newman*^2005^).

Power laws (see detail in (Clauset et al. 2009; Newman 2005; Virkar and Clauset 2005; 2014)) are widely used to represent scientometric distributions. In reality, however, certain studies of citation distributions have used various other functional forms to provide best approximations to as wide a variety of bibliometric data as possible (see, e.g., (Burrell 2014; Davies 2002; Golosovsky and Solomon 2012; Gupta et al. 2005; Laherrère and Sornette 1998; Radicchi et al. 2008; Redner 2005; Sangwal 2013; van Raan 2001)). Nevertheless, all the same, power laws had and still have a crucial part to play in scientometrics, not only because they are established but also because they are theoretically well-founded, for reasons arising from the generalized central limit theorem (Uchaikin and Zolotarev 2011), which has very considerable importance in probability theory.

One of the better models for the citation distribution is the Tsallis distribution (Anastasiadis et al. 2010; Bletsas and Sahalos 2009; Tsallis and de Albuquerque 2000; Wallace et al. 2009)

(3) $$  (q < 2)(\lambda > 0) \colon f(z) \propto {\lambda}(2 - q)e_{q}(- \lambda z),  $$

where

(4) $$  e_{q}(z) = \left\{ \begin{aligned} &\exp(z)\qquad\quad \,\,\,\, \text{if}\ q = 1, \\ &\left(1 + {\rho} z\right)^{\frac{1}{\rho}} \qquad \text{if}\ \left((1 + {\rho} z) > 0\right)\bigwedge (q \neq 1) \\ &0,\qquad\qquad\quad\,\,\, \text{otherwise} \end{aligned} \right.  $$

is the *q*-exponential. (Here we use the symbol *ρ* to denote (1−*q*)). The *q*-exponential can also be defined by the following equation describing the (temporal) nonlinear relaxation of a system from an unstable point:

$$ \frac{d\mathbf{e}}{dt} = - \mathbf{e}^{q} $$

with **e** given by *e*_*q*_(−*t*). The meaning of this statement is quite understandable.

In turn, the Tsallis distribution (3) may be regarded as a special case of the generalized Pareto distribution (GPD for short) (Bermudez and Kotz 2010)

$$(z\geq\mu)(\xi \neq 0)\colon f(z) = \frac{1}{\sigma}\left(1 + \frac{\xi(z - \mu)}{\sigma}\right)^{\left({-\frac{1}{\xi} - 1}\right)}, $$

where *μ*=0, $\xi = \frac {q - 1}{2 -q}$, *σ*=0. We also can say that the random variable (or, in abbreviated form, RV) *Z* has a GPD if (essentially) the RV *Z* can be expressed as *k*+*ϕ*(1−*U*)^−*δ*^, where *U* is a standard uniform RV. We intend to show here a specific but common heuristic model that can be adopted to generalize the GPD.

The practice of citations evolves over time. We can conceive of the process of citation as a way of tracking discrete social acts. Time lends citations direction and meaning (see (Bouabid 2011; Burrell 2002, 2014; Eom and Fortunato 2011; Glänzel 2007; Hsu and Huang 2011; Radicchi et al. 2012; Redner 2005; Simkin and Roychowdhury 2012; Wang et al. 2013) for more details). However, when we analyze bibliometric data sets, we may interpret citations not as a series of discrete acts but rather as a statistical regularity which can be expressed in the language of timeindependent PDs. While the very meaning of the RV *Z* is difficult to represent in terms of the PD, it acquires a direct intuitive sense in terms of the terminal time formalism, which is developed in a systematic way (a nice general reference book for Markov processes is (Sharpe 1988)). The formal solution may consist in making the terminal time, or the lifetime, the main source of information of the RV *Z*. For a given process of citation, the terminal time is random. To define a realization (of the Markov process of citation) we must describe the corresponding conditional probability *W*. There is a natural way to associate with the terminal time problem the conditional probability *W* that the Markov process of citation does not stop during the fixed time interval, given that all phenomena, connected with this process during the same time interval, are known. It is proved (cf. (Sharpe 1988, [Chap. VII])) that *W* is connected with (nonnegative and right-continuous with respect to time) additive functionals of the initial Markov process of citation. We recall that an additive functional of a Markov process *X* is a map which associates with each interval of time [*s*,*t*] a RV ${a_{t}^{s}}$, where ${a_{t}^{s}}$ depends only on the evolution of *X* during the time [*s*,*t*], and also the condition ${a_{t}^{s}} + a_{\tau }^{t}=a_{\tau }^{s}$ holds for arbitrary *t*∈[*s*,*τ*].

The approach proposed in this paper consists in letting the probability *W* play a crucial part by summarizing enough information about social citation system. As a rough guide, we suppose that the RV *Z* depends on time through the probability *W*.

The issue addressed in this paper is the development of a citation distribution that can be characterized in terms of the conditional probability *W* (given the total information concerning the performance of the process of citation for time *t*) that the Markov process of citation is of a duration longer than the time *t*. For the moment, we are not concerned with the explicit time dependence of the citations. In this paper, we assume that the RV *Z* is a function

(5) $$  \varphi(w) := \left\{ z\in Z\colon \exists w \ \left((w \in W) \wedge \left(z = \varphi(w)\right)\right)\right\},  $$

which we have yet to treat. We shall adopt an “asymptotic” point of view. We shall only be interested in the relation *φ*(·):*W*→*Z* that holds between *W* and *Z* at large times.

The proposed approach is based on the concept of the approximate invariance of the function (*w*∈[0,1]):*w*↦*φ*(*w*) by a translation of *w*, i.e., we claim that *φ*(*w*+·)≈ *φ*(*w*)*φ*(·). The considered heuristic model for the Markov process of citation is formulated as the inhomogeneous Choquet–Deny convolution equation (we shall use the abbreviated notation ICDCE) whose form is apparently determined by the approximate translation invariance. The solution of this equation gives the Wakeby distribution (WD) for citations of scientific papers. Until now, the WD has not been among the distributions employed to model observed bibliometric data.

The rest of this paper is organized as follows. The main result regarding our proposed model and its analytical solution is presented in the 2nd section. The empirical verification is provided in the 3rd section. Finally, concluding remarks are presented in the 4th section. The Appendix 1 introduces certain necessary definitions and reviews results that are needed in the rest of the paper.

## Model of citation distribution

The model *w*↦*φ*(*w*) can work reasonably well in scientometrics for social citation systems that are either sufficiently “ordered” or sufficiently “disordered”. In the limit of a large social citation system, we may at least assume that social citation system can be decomposed into a “structured” subsystem and a “stochastic” subsystem. For sake of concreteness, let us depart from the hypothesis that social citation system includes two types of subsystems whose nature is quite different. One of them could be identified as a social network, the other as a scientific market:

- The social network (sufficiently structured subsystem) is a polycentric complex of interrelated scholars.
- The scientific market (sufficiently stochastic subsystem) contains autonomous scholars who enter into the competition.
- The social network is characterized by structural cohesion, while the scientific market is actually an amorphous medium for sharing information resources.
- The evolution of the scientific market is of a stochastic nature.
- The social network corresponds to the notion of a dynamic system.
- The statistical properties of the citation distribution are partially determined by the nature of interactions between scientific market and social network.

Employing the previous notation, the postulated heuristic propositions, on the basis of which our model of the citation distribution is constructed, are as follows:

1. In the event horizon where the scientific market “lives”, it can be assumed that the function *φ*(*w*) in the expression (5) is invariant under translation of *w*
  (6) $$  \varphi(w+\cdot)=\varphi(w)\varphi(\cdot).  $$
2. In the event horizon of the social network the function *φ*(*w*) may be intuitively considered as the positive contraction semigroup *τ*(*w*) on a real one-dimensional Banach space generated by −*β*
  (7) $$  (\beta \in \mathbb{R})\colon \tau(w) = \exp(- \beta w).  $$
3. The social logic of the citation distribution is such that there is a two-way influence between the scientific market and the social network (Bourdieu 1975). However, in the limit of long time, social effects of the process of citation bring to screening “long-range” interactions. As a result, the subsystems in social citation system are almost independent and we obtain approximate translation invariance
  (8) $$  \varphi(w + \cdot) = \varphi(w)\varphi(\cdot) + r(w),  $$
  where *r*(*w*) indicates a remainder term.

In the framework of previously accepted propositions the following statements are considered:

- The simplest and most intuitive general approach to translate invariance is via convolution. Let *T*_*a*_ be the translation operator defined by *T*_*a*_*φ*(*w*)=*φ*(*w*+*a*). Translation invariance of the convolution (*φ*∗*χ*) means that the convolution with a fixed function *χ* commutes with *T*_*a*_, i.e.,
  $$ T_{a}\left(\varphi \ast \chi\right) = \left(T_{a}\varphi\right) \ast \chi = \varphi \ast \left(T_{a}\chi\right). $$
  It can involve explicitly the well-known Choquet–Deny convolution equation (CDCE for short, see (20)).
- By virtue of formula (7), whatever the precise form of *r*(*w*) may be it will give to (8) a contribution of the form
  $${\lim}_{w\uparrow{1}} r(w) = O\left(\exp(- \beta w)\right). $$
  This proposition corresponds to a functional equation that can be rewritten as the ICDCE (see (21)).

The translation invariance is an important concept, so it should be understood in a thorough manner. The probability *W*, of course, corresponds to terminal time, while the RV *Z* occurs at random in time. Since the RV *Z* in the scientific market should be independent of an arbitrary translation *a*, the constancy of termination rate of the process of citation take place in the scientific market. This is what we mean when we say that *φ*(*w*) has the translation invariant property (6) in the scientific market.

The motivation of the approximate translation invariance is to take the relation between the scientific market and the social network into consideration. In rough approximation, the scientific market and the social network can be considered as relatively independent. Consequently, their contributions to *φ*(*w*) are additive. Summing (6), and (7), we obtain (8), i.e., approximate translation invariance. The Eq. 8 therefore expresses some kind of linear superposition of the effect of the scientific market and the social network. This superposition is not valid in the general case.

To find the citation distribution that we seek, we will start off with certain well-known mathematical constructions. Let $\left (\Omega, \mathcal {F}, (\mathcal {F}_{t})_{t\in I}, \mathbf {P}\right)$ be a filtered probability space that satisfies the usual conditions (for details, see Chap. 1 of (Sharpe 1988)). In constructing a model of the citation distribution, we can imagine the social citation system as a normal Markov process *X*=(*X*_*t*_)_*t*∈*I*_ in a state space $(S,\mathcal {S})$. Insofar as our interest in the social citation system is confined to a few of its features, the Markov process-based model may be relevant in explaining the citation distribution. Further, we shall suppose that the experimentally observed Markov process $\tilde {X}$ is obtained from *X* by curtailment of its terminal time up to $\tilde {\zeta }$

$$(I \ni \tilde{\zeta}\colon\Omega \rightarrow \mathbb{R}_{+})(t<\tilde{\zeta})\colon \tilde{x}(t,\tilde{\omega}) = x(t,\omega). $$

Equivalently, the process $\tilde {X}$ is given by a truncation of the duration of the original process *X* such that the trajectories of *X* are terminated in a random manner. One can easily see that, for a proper choice of the filtered probability space $\left (\tilde {\Omega }, \tilde {\mathcal {F}}, {(\tilde {\mathcal {F}}_{t})}_{t\in I}, \tilde {\mathbf {P}}\right)$ and the state space $(S,\mathcal {S})$, the process $\tilde {X}$ is a subprocess of the process *X*. The duration of the processes *X* and $\tilde {X}$ are denoted by *ζ* and $\tilde {\zeta }$, respectively, and

$$ \left(\forall (s, x)\right)\colon \mathbf{P}_{s,x}\left(\tilde{\zeta} \leq \zeta\right) = 1. $$

The construction of such a subprocess is minutely described in (Sharpe 1988 [p. 65–74]).

Under appropriate assumptions, we can represent the Markov process $\tilde {X}$ using the concept of the multiplicative functional of the Markov process *X*. This approach is explained in detail in (Sharpe 1988, [p. 286–301]). Let us now introduce the contracting, multiplicative functional $(s\leq t\leq \infty)\colon {m_{t}^{s}}\colon I(\omega)\rightarrow (S,\mathcal {S})$ continuous from the right on *X*. It is proved (see Theorem 4 in (Gikhman and Skorokhod 2004, [p. 71–72])) that

(9) $$ \begin{aligned} &\left({\Omega^{s}_{t}}=\left\{\omega\colon {s,t}\in I(\omega)\right\} \right) \left(\mathrm{a.s.}\ {\Omega^{s}_{t}}, \mathbf{P}_{s,x}\right)\colon \\ &{m_{t}^{s}} = \tilde{\mathbf{P}}_{s,x}\left(\tilde{\zeta} > t \ \left\vert\, \left(\tilde{\mathcal{F}}^{s} \right)_{s\in I}\right.\right).  \end{aligned}  $$

Let ${a_{t}^{s}}\colon I(\omega)\rightarrow (S,\mathcal {S})$ be an additive functional, continuous from the right on *X*. The formulae ${m_{t}^{s}}=\exp (-{a_{t}^{s}})$, ${a_{t}^{s}}=-\ln {{m_{t}^{s}}}$ establish a one-to-one correspondence between ${a_{t}^{s}}$ and ${m_{t}^{s}}$ (Gikhman and Skorokhod 2004 [p. 64]). It follows in the usual way that,

(10) $$  \left(\mathrm{a.s.}\ \Omega_{t}, \mathbf{P}_{t}\right) \colon \tilde{\mathbf{P}}_{s,x}\left(\tilde{\zeta} > t \ \left\vert\, \left(\tilde{\mathcal{F}}^{s}\right. \right)_{s\in I}\right) = \exp\left(-{a_{t}^{0}}\right).  $$

In the expression (10), of the quantity

$$\tilde{\mathbf{P}}_{s,x}\left(\tilde{\zeta} > t \ \left\vert\, \left(\tilde{\mathcal{F}}^{s}\right. \right)_{s\in I}\right) $$

can be interpreted as the conditional probability *W* that the trajectory *x*(*τ*) does not terminate during the time interval [0,*t*]. Moreover, to simplify the argument, we set

$$\left(\forall t\in I(\omega)\right)\colon {a_{t}^{0}} = \vartheta t. $$

Then we immediately verify that,

(11) $$ \tilde{\mathbf{P}}_{x}\left(\left. \tilde{\zeta} > t \right\vert (\mathcal{F}_{t})_{t\in I}\right) = \exp(-\vartheta t),   $$

where $\tilde {\mathbf {P}}_{x}\left (\left. \tilde {\zeta } > t \right \vert (\mathcal {F}_{t})_{t\in I}\right)$ holds for the conditional probability *W* that the process of citation is of duration longer than *t*:

$$W \equiv \tilde{\mathbf{P}}_{x}\left(\left. \tilde{\zeta} > t \right\vert (\mathcal{F}_{t})_{t\in I}\right). $$

We assume without essential loss of generality that under a suitable normalization, the RV *W* has a standard exponential distribution. With the inverse method, we have

(12) $$  W = -\ln U.  $$

It follows from the above that the properties of the distribution **P**_*Z*_(*z*) depend on *w*. To be thorough, we must note that the distribution **P**_*Z*_(*z*) is defined on the probability space $\left (\mathfrak {Z},\mathcal {B}(\mathfrak {Z}),\mathbf {P}_{Z}\right)$. Obviously, the connections between the Markov process *X* and the distribution **P**_*Z*_(*z*) may be based on the concept of the conditional probability *W*. A somewhat unrealistic, but simple, schematic idea of these connections is given by the equality

(13) $$ \begin{array}{c} \left(\!\left(\forall{z}\in{\mathbb{R}_{+}}\right)\left({\left\{\mathfrak{z}\colon Z(\mathfrak{z})\leq z\right\}}\in{\mathcal{B}(\mathfrak{Z})}\right)\colon Z\colon \mathfrak{Z}\rightarrow\mathbb{R}_{+}\!\right) \\ \left(\varphi(w)\colon\mathbb{R}_{+}\rightarrow\mathbb{R}_{+}\right)\colon {z}={\varphi(w)},  \end{array}  $$

where, as in Appendix 1, *φ*(*w*) is locally integrable (with respect to the Lebesgue measure *Λ*). However, the function *φ*(*w*) is not yet completely defined. In fact, the general problem of studying the form of *φ*(*w*) can be reduced to the case in which this function satisfies certain extra conditions. One can attempt to define *φ*(*w*) implicitly by some functional equation rather than by direct definitions. In particular, the general form of *φ*(*w*) may be derived uniquely from its invariance.

For the purpose of our study, based upon the denotation introduced in Appendix 1, let *μ*^*n*^ be the *n*-fold convolution of *μ*, and let *φ*(*w*) be a nontrivial positive solution of the ICDCE (21). Observe first that from the paper of (Gu and Lau 1984), we know that for a.a. (mod *Λ*)$w\in \mathbb {R}_{+}$, we have the relation

$$\begin{aligned} \varphi(w) &= {\lim}_{n\to\infty}\int_{\mathbb{R}_{+}}\varphi(w + v)\, \mu^{n}\, (dv) \\ &\quad+ \sum_{n=0}^{\infty}\int_{\mathbb{R}_{+}}\varphi(w + v)r(w + v)\, \mu^{n}\, (dv). \end{aligned} $$

Suppose *μ* is a non-probability measure. If we take *μ* without requiring $\int _{\mathbb {R}_{+}}\mu (dv) = 1$ and, *mutatis mutandis*, use the arguments employed by (Gu and Lau 1984), we obtain the following expression for *φ*(*w*):

(14) $$  \varphi(w) \propto \kappa_{1}\exp(\delta w) + \kappa_{2}\exp(-\beta w),  $$

where *κ*_1_ and *κ*_2_ are constants. It should be mentioned that the definition (13) allows us to write the function *φ*(*w*) in an explicit form of the RV *Z*

(15) $$  Z \propto \kappa_{1}\exp(\delta w) + \kappa_{2}\exp(-\beta w).  $$

This expression is the relation we were seeking between the quantities we were interested in, *Z* and *W*. As could be expected, the RV *Z* contains two parts: one corresponds to the incident stream of citations, the other to the scattered stream of citations.

To extract the implications of (15), it is convenient to represent the RV *W* in terms of the uniform RV *U*. Now, if we recall the Eq. 12, the expression (15) can be straightforwardly rewritten as

(16) $$  Z \propto \kappa_{1} U^{-\delta} + \ \kappa_{2} U^{\beta}.  $$

The study of the relation (16) makes it possible to obtain the PD of the RV *Z*. Motivated by the approximate translational invariance of *z* with respect to the probability *w* that the process of citation does not terminate, we suggest that this model is appropriate to provide a phenomenologically relevant picture of the citation distribution. Finally, starting from the statistical considerations connected with a common and convenient choice of distribution function (Johnson et al 2010, [Chap. 12]), a natural modification of the relation (16) can be written in the form

(17) $$  Z = \upsilon(1 - U)^{-\delta} - \ \theta (1 - U)^{\beta} + k.  $$

The formula (17) defines the distribution, which is called the WD (Johnson et al. 2010, [p. 44–46]). This distribution was established by H. A. Thomas (Houghton 1978) (who lived on Wakeby pond on Cape Cod, Massachusetts) for hydrological data case studies (Griffiths 1989; Hosking and Wallis 2005). We stress that the explicit formula for the PDF of *Z* is not generally available.

For the sake of being definite, it would be better to rewrite (17) using the following notation

$$\upsilon = \gamma/\delta,\; \theta = \alpha/\beta,\; k = \xi + \theta - \phi. $$

Suppose all parameters *α*, *β*, *γ*, *δ*, *ξ* are continuous. Then, the WD becomes

(18) $$  Z = \xi +\frac{\alpha}{\beta}\left(1 - (1 - U)^{\beta}\right) - \frac{\gamma}{\delta}\left(1 - (1 - U)^{-\delta}\right).  $$

It is readily seen that the WD has three disposable shape parameters, one location parameter and one scale parameter. Under the following conditions:

$$\begin{aligned} (\alpha \neq 0) & \vee (\gamma \neq 0), \\ (\beta + \delta > 0) & \vee (\beta = \gamma = \delta = 0), \\ (\alpha = 0) & \Rightarrow (\beta = 0), \\ (\gamma = 0) & \Rightarrow (\delta = 0), \\ (\gamma \geq 0) & \wedge (\alpha + \beta \geq 0) \end{aligned} $$

the Eq. 18 has a unique solution on dom *Z*; here

$$ \text{dom}~Z =\left\{ \begin{aligned} &[\xi, \infty)\qquad\qquad\qquad\,\, \text{if} \ (\delta \geq 0) \wedge (\gamma >0), \\ &\left[\xi, \xi + \frac{\alpha}{\beta} - \frac{\gamma}{\delta}\right] \qquad \text{if} \ (\delta > 0) \vee (\gamma = 0). \end{aligned} \right. $$

The WD in (18), when *α*=0 or *γ*=0 reduces to the GPD. The Eq. 18 is not very tractable for analysis but can yield efficient algorithms for the numerical simulation of the WD.

Nearly all the papers that deal with inference for the WD are based on the theory of *L*-moments (Hosking 1990, 2006; Hosking and Wallis 2005). The free software statistical environment R contains functions to estimate the parameters of the WD from the data (see, e.g., (Asquith 2011), and packages ‘lmom’, ‘lmomco’).

## Illustration

To demonstrate the applicability of the proposed heuristic model, we evaluate the goodness-of-fit of the WD to two bibliometric datasets.

### Data sets

This study is based on the citation distribution of papers published by the American Physical Society (APS), the American Mathematical Society (AMS), the European Mathematical Society (EMS), and the Institute of Physics (IOP) (see the list of journals in Appendix 2) in the years 1980 —2008 and indexed in Thomson Reuters Journal Citation Reports, Science Edition 2012. The data on citations was obtained from the Thomson Reuters Web of Science Core Collection. The data on citations of papers of APS, AMS and EMS were obtained in December 2013. The data for IOP were received in April 2014. The number of citations *z* is counted as the total number of times a paper appears as a reference of a more recently published paper indexed in the Web of Science Core Collection.

Two sets of bibliometric data are tested in the study:

- The first set contains papers published by APS, AMS, and EMS. There are 10,043,731 citations among 356,287 papers.
- The second set consists of 233,570 papers published by IOP. This dataset includes 5,885,458 citations.

### Empirical results

**Figure 1:**
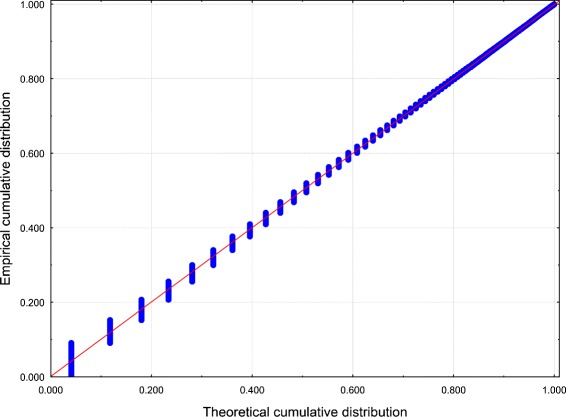
**Probability – Probability plot of** ***Z*** **for Dataset 1.** Distribution: WD.

**Figure 2:**
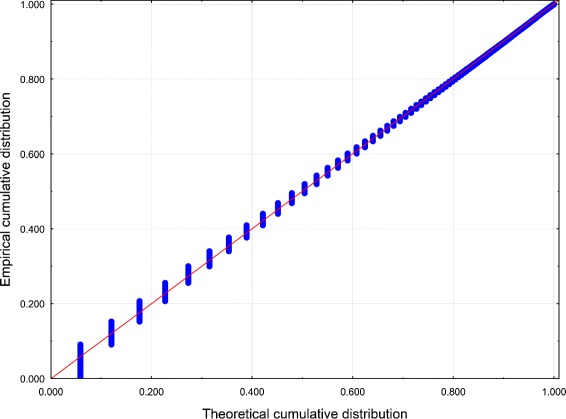
**Probability – Probability plot of** ***Z*** **for Dataset 1.** Distribution: GPD.

**Figure 3:**
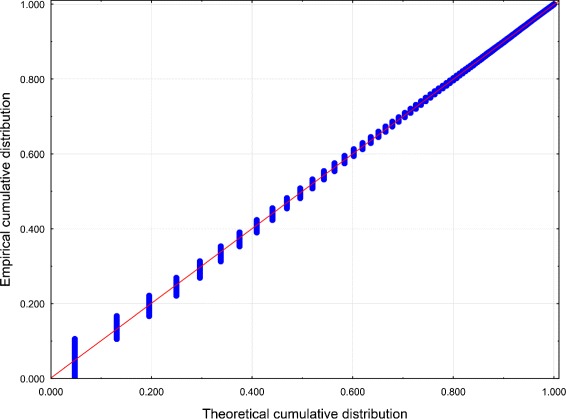
**Probability – Probability plot of Z for Dataset 2.** Distribution: WD.

**Figure 4:**
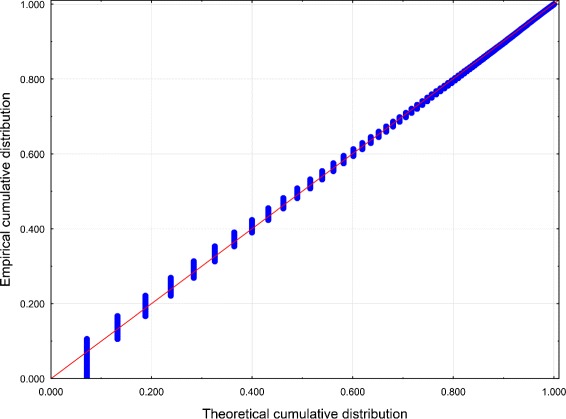
**Probability – Probability plot of** ***Z*** **for Dataset 2.** Distribution: GPD.

Best-fit PDs for both data sets were performed using the Mathwave EasyFit 2014 data analysis software. The 63 PDs were automatically fitted to the empirical distributions of the data sets. The Kolmogorov–Smirnov test and the Anderson–Darling test were performed to assess goodness-of-fit, and the PDs were ranked according to the goodness-of-fit. The values of the test statistics for the top 5 PDs are reported in Tables 1 and 2 (see also Figures 1, 2, 3 and 4).

**Table 1:** **Goodness of fit — Summary for Dataset 1**

**Distribution**	**Kolmogorov – Smirnov**		**Anderson – Darling**	
	**(** ***p*** **=0** ***.*** **00205,** ***α*** **=0** ***.*** **1)**		**(** ***p*** **=1** ***.*** **9286,** ***α*** **=0** ***.*** **1)**	
	**Statistic**	**Rank**	**Statistic**	**Rank**
WD	0.05036	1	785.96	1
GPD	0.05818	2	878.3	2
Gen. Extreme Value	0.0861	3	2359.3	3
Pareto 2	0.09083	4	47317.0	6
Phased Bi-Exponential	0.09143	5	53274.0	8

**Table 2:** **Goodness of fit — Summary for Dataset 2**

**Distribution**	**Kolmogorov – Smirnov**		**Anderson – Darling**	
	**(** ***p*** **=0** ***.*** **00253,** ***α*** **=0** ***.*** **1)**		**(** ***p*** **=1** ***.*** **9286,** ***α*** **=0** ***.*** **1)**	
	**Statistic**	**Rank**	**Statistic**	**Rank**
WD	0.05845	1	655.6	1
GPD	0.07127	2	796.09	2
Gen. Extreme Value	0.09249	3	1817.5	3
Gen. Logistic	0.09848	4	2022.5	4
Phased Bi-Exponential	0.10584	5	37917.0	10

Comparing the obtained values and goodness-of-fit statistics given in the Tables, it will be seen that the WD offers a greater level of accuracy than the other PDs considered.

## Discussion

We conclude that the WD is in some sense the best PD to adequately fit the examined bibliometric data sets.

It should be clear that the proposed heuristic approach is only a phenomenological model of the citation distribution. The Eq. 11 has not been derived yet but has rather been injected into the model. The vector of parameters (*α*,*β*,*γ*,*δ*,*ξ*), which fixes the WD, is assumed to be given. We can say that the formula (17) does not reproduce the exact citation distribution. We should rather view the expression (17) as an approximate representation, in which the fine details of the citation distribution have been rounded up for clarity. Nevertheless, discrepancies with observation may be caused by errors in data collection or by random influences, which will be explained later. Also, there may be many still unknown secondary effects that could change the shape of the citation distribution. But it does not detract from the consistency or the cognitive value of the mathematical model. The proposed heuristic model of the citation distribution may be considered as a potentially useful amalgamation of mathematical abstraction and scientometric intuition.

## Appendixes

### Appendix 1. Mathematical preliminaries to model development

In the context of this paper we are interested in mathematical formulations. Therefore, we briefly indicate here how the function *φ*(·) can be treated mathematically.

Let *μ* and *ν* be regular Borel measures on a locally compact Abelian group *G* with a countable basis. The convolution equation *μ*=*μ*∗*ν* on *G* was first thoroughly investigated by (Choquet and Deny 1960). The integral representation of unbounded solutions was generalized by (Deny 1959). For the sake of completeness, we introduce the following notation:

- $\psi \colon G \rightarrow \mathbb {R}_{+}$: the real-valued non-negative function;
- $C(G,\mathbb {R}_{+})$: the space of continuous functions from *G* to $\mathbb {R}_{+}$;
- *μ*: the Radon measure on the Borel *σ*-field $\mathcal {B}(G)$ that is generated by *G*;
- *Λ*: the Lebesgue measure;
- *Ψ*: the space of all real-valued non-negative functions $\psi (\cdot)\colon G \rightarrow \mathbb {R}_{+}$ such that
  (19) $$  (\forall x \in G)\left(\psi(\cdot)\in C(G,\mathbb{R}_{+})\right)\colon \psi(x + y) = \psi(x)\psi(y).  $$

The space *Ψ* is, by construction, a locally compact space with the topology of uniform convergence on compact sets. We define the subset *Ψ*_*μ*_⊂*Ψ* as follows

$$ \begin{aligned} &(\forall x \in G)\left(\psi(\cdot)\in C(G,\mathbb{R}_{+})\right)\colon \\ &{\Psi_{\mu}}{:=} \left\{\!\psi(\cdot)\colon\left(\psi(\cdot)\in\Psi\!\right)\!\bigwedge\! \left(\int_{G}\psi(x)\, \mu\, (dx)=1\!\right)\!\right\}. \end{aligned} $$

From the definition, *Ψ*_*μ*_ is a Borel subset of *Ψ*. In addition, let *G* itself be the smallest closed subgroup of *G* that contains supp(*μ*).

The generalized version of the Deny’s theorem is the following. When the real-valued non-negative continuous function $\phi (\cdot)\colon G\rightarrow \mathbb {R}_{+}$ satisfies the Choquet–Deny convolution equation:

(20) $$   (\forall x \in G)\left(\phi(\cdot)\in C(G,\mathbb{R}_{+})\right)\colon \phi(x) = \int_{G}\phi(x + y)\, \mu\, (dy),  $$

then there exists a unique measure *ν*_*ϕ*_ on *Ψ*_*μ*_ such that

$$ (\forall x \in G)\left(\phi(\cdot)\in C(G,\mathbb{R}_{+})\right)\colon \phi(x) = \int_{\Psi_{\mu}}\psi(x)\, \nu_{\phi}\, (d\psi). $$

For an extensive discussion of the whole problem, the reader is referred to (Lukec̆s et al. 2010, [Chap. 3]). The CDCE (20) and its ramifications occupy a central place in our study.

It should be noted that, according to (Deny 1959), if *μ* is a probability measure, then every bounded solution of (20) reduces to a constant.

In the case $G = \mathbb {R}_{+}$, *μ* is assumed to be non-arithmetic such that *μ*(*∅*)<1, and *ϕ*(·) is assumed to be non-negative, real-valued and locally integrable with respect to the *Λ* function (ignoring the trivial case of *ϕ*(·)=0 a.e. (mod *Λ*)) such that it satisfies a.a. (mod *Λ*) to the CDCE (20).

As a corollary of Deny’s theorem, Lau and Rao provided the following theorem, specifying the above result: If a nontrivial solution for *ϕ*(·) exists, then it is of the form

$$ \left(\mathrm{a.e.}\!\!\!\!\pmod{\Lambda} \ x \geq x_{\min}\right)\colon \phi(x) = p(x)\exp(cx), $$

where the relation

$$ \left(\forall u \in \text{supp} (\mu)\right)\colon p(\cdot + u) = p(\cdot) > 0 $$

is fulfilled with *c* such that

$$\begin{aligned} &(c\in \mathbb{R})\left(\mathrm{a.e.}\!\!\!\!\pmod{\Lambda} \ \forall x\in \mathbb{R}_{+}\right)\colon \\ &\int_{\mathbb{R}_{+}}\exp(cx)\, \mu\, (dx) = 1. \end{aligned}  $$

The proof of this theorem can be found in (Lau and Rao 1982).

The inhomogeneous Choquet–Deny convolution equation (ICDCE)

(21) $$ \begin{aligned} &\left(\mathrm{a.e.}\!\!\!\!\pmod{\Lambda} \ \forall x\in \mathbb{R}_{+}\right)\colon \\ &\phi(x) = \int_{\mathbb{R}_{+}}\phi(x + y)\, \mu\, (dy) + r(x), \end{aligned}  $$

where |*r*(*x*)|≤*κ* exp(−*βx*) is an “error term”, is a generalization of the Eq. 20 given by Shimizu. The solutions of the ICDCE on $\mathbb {R}_{+}$ were considered by (Shimizu 1980)and by (Gu and Lau 1984).

### Appendix 2. List of journals

- American Physical Society
  1. Physical Review A
  2. Physical Review B
  3. Physical Review B
  4. Physical Review C
  5. Physical Review D
  6. Physical Review E
  7. Physical Review Letters
  8. Physical Review Special Topics Accelerators And Beams
  9. Physical Review Special Topics Physics Education Research
  10. Physical Review X
  11. Reviews of Modern Physics
- American Mathematical Society
  1. Bulletin of American Mathematical Society
  2. Journal of the American Mathematical Society
  3. Mathematics of Computation
  4. Memoirs of the American Mathematical Society
  5. Proceedings of the American Mathematical Society
  6. St. Petersburg Mathematical Journal
  7. Transactions of the American Mathematical Society
- European Mathematical Society
  1. Commentarii Mathematici Helvetici
  2. Groups Geometry and Dynamics
  3. Interfaces and Free Boundaries
  4. Journal of Noncommutative Geometry
  5. Journal of the European Mathematical Society
  6. Portugaliae Mathematica
  7. Rendiconti Lincei —Matematica e Applicazioni
  8. Revista Matematica Iberoamericana
  9. Zeitschrift für Analysis und Ihre Anwendungen
- Institute of Physics
  1. Astronomical Journal
  2. Astrophysical Journal
  3. Astrophysical Journal Letters
  4. Astrophysical Journal Supplement Series
  5. Bioinspiration Biomimetics
  6. Biomedical Materials
  7. Chinese Physics B
  8. Chinese Physics Letters
  9. Classical and Quantum Gravity
  10. Communications in Theoretical Physics
  11. Environmental Research Letters
  12. European Journal of Physics
  13. Fluid Dynamics Research
  14. Inverse Problems
  15. Journal of Breath Research
  16. Journal of Cosmology and Astroparticle Physics
  17. Journal of Geophysics and Engineering
  18. Journal of Instrumentation
  19. Journal of Micromechanics and Microengineering
  20. Journal of Neural Engineering
  21. Journal of Physics A Mathematical and Theoretical
  22. Journal of Physics B Atomic Molecular and Optical Physics
  23. Journal of Physics: Condensed Matter
  24. Journal of Physics D Applied Physics
  25. Journal of Physics G Nuclear and Particle Physics
  26. Journal of Radiological Protection
  27. Journal of Statistical Mechanics Theory and Experiment
  28. Laser Physics
  29. Laser Physics Letters
  30. Measurement Science Technology
  31. Metrologia
  32. Modelling and Simulation in Materials Science and Engineering
  33. Nanotechnology
  34. New Journal of Physics
  35. Nonlinearity
  36. Physica Scripta
  37. Physical Biology
  38. Physics in Medicine and Biology
  39. Physics World
  40. Physiological Measurement
  41. Plasma Physics and Controlled Fusion
  42. Plasma Science Technology
  43. Plasma Sources Science Technology
  44. Reports on Progress in Physics
  45. Semiconductor Science and Technology
  46. Smart Materials and Structures
  47. Smart Materials Structures
  48. Superconductor Science Technology
